# Association between Albumin–Globulin Ratio and Mortality in Patients with Chronic Kidney Disease

**DOI:** 10.3390/jcm8111991

**Published:** 2019-11-15

**Authors:** Pin-Pin Wu, Yao-Peng Hsieh, Chew-Teng Kor, Ping-Fang Chiu

**Affiliations:** 1 Department of Internal Medicine, Changhua Christian Hospital, Changhua 50006, Taiwan; 2 Division of Nephrology, Department of Internal Medicine, Changhua Christian Hospital, Changhua 50006, Taiwan; 3 School of Medicine, Kaohsiung Medical University, Kaohsiung 80708, Taiwan; 4 School of Medicine, Chung Shan Medical University, Taichung 40201, Taiwan; 5 Department of Recreation and Holistic Wellness, MingDao University, Changhua 52345, Taiwan

**Keywords:** albumin-globulin ratio, cardiovascular disease, chronic kidney disease, inflammation, mortality

## Abstract

Background: Malnutrition and inflammation are highly prevalent and tightly regulated with each other in chronic kidney disease (CKD) patients. Inflammation can lead to malnutrition in patients with sufficient nourishment, while malnutrition may also induce an inflammatory response. This study investigated whether the albumin-globulin ratio (AGR) can predict the mortality risk in CKD patients. Methods: We enrolled 956 stage 3–5 CKD patients retrospectively at a medical center. Patients’ baseline characteristics including demographics, laboratory data, pharmacotherapy, and comorbidities were collected for statistical adjustments. The study patients were stratified into three AGR groups according to similar magnitudes of hazards for mortality as follows: low AGR group, AGR ≤ 1.0; moderate AGR group, 1.1 ≤ AGR < 1.3; high AGR group, AGR ≥1.3. Multivariate Cox proportional hazard analysis was performed to evaluate the association of the AGR with the study outcomes, including overall and cardiovascular disease (CVD) mortality. Results: During a median follow-up duration of 2.44 years, 108 (11.3%) deaths were recorded and 50 patients died from CVD. In adjusted model 1, the moderate AGR group was associated with hazard ratios (HR) of 0.57 (95% CI = 0.36–0.90, *p* = 0.016) and 0.52 (95% CI = 0.28–0.98, *p* = 0.043) for all-cause and CVD mortality compared with the low AGR group, respectively. The high AGR group was associated with HRs of 0.49 (95% CI = 0.27–0.90, *p* = 0.021) and 0.27 (95% CI = 0.1–0.74, *p* = 0.01) for all-cause and CVD mortality compared with the low AGR group, respectively. Similar results were obtained in the adjusted model 2 (inverse probability of the group weighted Cox model). In addition, the association between the AGR and mortality risk remained significant when the AGR was treated as a continuous variable. Conclusion: AGR is a significant biomarker predicting overall and cardiovascular mortality risk independent of various important factors amongst stage 3–5 CKD patients. We suggest that the AGR may be a simple and inexpensive measurement for detecting CKD patients at risk of mortality.

## 1. Introduction

Chronic kidney disease (CKD), characterized by the retention of uremic toxins due to the progressive decline in kidney function, is a growing health problem worldwide and is associated with significant morbidity and mortality. The number of CKD patients has substantially increased in the past three decades due to an aging population with multiple comorbidities [1]. CKD affects about 11.5% of the general population [2]. The prevalence of CKD has even reached 35.8% in patients older than 64 years [3]. Diabetes mellitus (DM) is the leading cause of CKD, and approximately 40% of DM patients have CKD in the United States [4]. Most of the diabetic patients with CKD ultimately died of cardiovascular disease (CVD) [5]. Therefore, CKD is well recognized as a CVD equivalent and should be regarded as a priority for public health.

Malnutrition and inflammation are highly prevalent and tightly regulated with each other in CKD patients [6]. Inflammation can lead to malnutrition in patients with sufficient nourishment, while malnutrition may induce an inflammatory response [7]. In general, serum albumin is used to reflect the degree of nutritional status, whereas serum globulin is cited to assess the severity of chronic inflammation. As with most parameters, both albumin and globulin concentrations are affected by various factors, e.g., the volume status of body fluid. Recently, the albumin-globulin ratio (AGR) emerged as a novel prognosticator. Prior investigations reported the preoperative AGR as a simple and valuable predictor for evaluation of prognosis in several cancers, including colorectal cancer, nasopharyngeal cancer, lung cancer, and breast cancer [8,9,10,11].

Despite the advancing improvements in managing patients with CKD, the high mortality still needs to be improved. Thus, the discovery of prognostic markers for mortality, which are easy to assess and accessible in clinical practice, can help with decision making in the management of CKD patients. Although the roles of chronic inflammation and malnutrition in CKD are well known, there is currently very limited evidence on the predictive role of AGR for mortality risk in CKD patients. Thus, we aim to conduct this study to determine whether AGR would predict patient survival in patients with stage 3–5 CKD.

## 2. Materials and Methods

### 2.1. Participants and Study Design

We conducted a retrospective investigation at a medical center in Central Taiwan. Patients who joined the integrate pre-dialysis CKD care program at the nephrological outpatient clinic between 1 January 2011 and 31 July 2016 were screened for the eligibility of this study and followed until 31 July 2017. Kidney function, using estimated glomerular filtration rate (eGFR), was determined using the simplified 4-variable equation from the Modification of Diet in Renal Disease (MDRD) study: estimated GFR mL/min per 1.73 m^2^ = 186 × serum creatinine^−1.154^ × age^−0.203^ × 0.742 (if female patient) × 1.212. CKD stages were classified based on the criteria from the National Kidney Foundation Kidney Disease Outcomes Quality Initiative (K/DOQI). Patients were classified as stage 3, 4, and 5 CKD if they had an eGFR (ml/min per 1.73 m^2^) of 30–59, 15–29, and <15, respectively. Finally, a cohort of 956 patients with stage 3–5 CKD were suitable for the study after excluding stage 1–2 CKD patients and those lost to follow-up within three months. This retrospective study was approved by the Institutional Review Board of Changhua Christian Hospital (CCH-IRB-190606) and carried out in adherence with the ethical principles expressed in the declaration of Helsinki for medical research. Due to the non-intrusiveness and anonymity for such retrospective work, we were granted a waiver of informed consents from the Institutional Review Board.

### 2.2. Collection of Demographic, Medical, and Laboratory Data and Study Outcomes

Baseline patients’ information and data were gathered from the established electronic dataset and by reviewing the medical charts as needed. Baseline clinical data included sex, age, body mass index (BMI), smoking status, alcohol status, medical history, medication use, and laboratory data. Both smoking and alcohol drinking status were categorized as never, current, or ever. The collected comorbidities included diabetes mellitus (DM), hypertension, cardiovascular disease (CVD), gout, and chronic lung disease, while the pharmacotherapy consisted of angiotensin-converting enzyme inhibitor (ACEI), angiotensin II receptor blockers (ARB), lipid-lowering agents (statin), vitamin D, calcium supplements, calcium channel blockers (CCB), erythropoiesis-stimulating agents (ESA), non-steroidal anti-inflammatory drugs (NSAID) and pentoxyfylline. Blood and urine tests comprised of blood urea nitrogen (BUN), creatinine, albumin, AGR, white blood cell counts (WBC), hemoglobin, hemoglobin A1c (HbA1c), sodium, potassium, cholesterol, triglyceride, uric acid, calcium, phosphate, and urine protein-to-creatinine ratio, which was used to quantify the daily urinary protein excretion. AGR was calculated using the following formula: AGR = albumin/(total proteins-albumin). All patients were followed up at the nephrology outpatient clinic periodically for treatment of CKD-related complications and routine blood tests. The leading cause of mortality in our patients was CVD. The outcomes of this study were all-cause mortality and CVD mortality.

### 2.3. Statistical Analysis

The study patients were stratified into three AGR groups according to similar magnitudes of hazards for mortality as follows: low AGR group, AGR ≤1.0; moderate AGR group, 1.1≤ AGR <1.3; high AGR group, AGR ≥1.3 ([12]; Supplementary Figure S1). Descriptive statistics of baseline patient characteristics for the three AGR patient groups were shown as a number (percentage) for categorical variables, mean ± standard deviation (SD) for continuous variables of normal distribution, or median (interquartile range, IQR) of non-normal distribution. The presence of a normal distribution was determined by the Kolmogorov-Smirnov test. Differences in distribution of variables among the three AGR groups were compared using Chi-square tests or a one-way analysis of variance (ANOVA), as appropriate. Survival comparisons in terms of overall mortality and CVD mortality were analyzed by the Kaplan-Meier method and the significance was determined by the log-rank test.

To test the independent association between the AGR and clinical outcomes, we performed two statistical regression models. We first calculated the propensity score for adjustments and the variables for the construction of the propensity score included gender, age, BMI, smoking and alcohol consumption status, the comorbid conditions, and medication therapy. In model 1, the association of AGR groups with study outcomes was evaluated by using the Cox proportional hazard model with adjustments for the propensity score and the laboratory data. On the other hand, we performed inverse probability of group-weight (IPW) Cox analysis to assess the AGR-mortality relationship in model 2. IPW was generated from the generalized boosted regression and was fit to control the imbalances of the covariates distribution and eliminate residual confounding between treatment groups. The balance of covariates was achieved if the maximum standardization difference was <0.1. The variables to be tested for the balance achievement after IPW included sex, age, BMI, smoking and alcohol status, comorbidities, and medication use. Thus, model 2 was constructed using the IPW Cox model which incorporated laboratory data and variables with a maximum standardization difference of >0.1. Hazard ratio (HR) and 95% confidence interval (CI) were shown for the risk of study outcomes associated with the AGR.

Two levels of sensitivity tests were conducted to assess the robustness of our study. First, the HR of the AGR associated with clinical outcomes of interest was calculated with the AGR treated as a continuous variable in Model 1. Second, we repeated the model 2 analysis with the AGR treated as a continuous variable. We also tested the potential interactions between the AGR and mortality in subgroups of patients stratified by sex, age, and comorbid diseases (diabetes mellitus, hypertension, gout, chronic lung disease, and CVD). R language and SPSS statistical software, version 20.0 (SAS Institute Inc., Cary, NC) were used to analyze the data, and a statistical significance was considered at a two-tailed *p* value < 0.05.

## 3. Results

### 3.1. Patients’ Baseline Characteristics

A total of 956 patients with pre-dialysis stage 3–5 CKD (529 men and 427 women) were enrolled as the study cohort. The mean age was 67.8 ± 12.9 years and the median follow-up duration was 2.44 (1.51–4.02) years for the entire population. The entire cohort was stratified into three groups based on the similar magnitude of hazard for mortality. There were 138, 535, and 283 patients in the low AGR, moderate AGR, and high AGR groups, respectively. The clinical characteristics of these study groups were compared and shown in Table 1. Patients in the low AGR group were likely to be women, older, non-alcohol drinkers, and had a higher BMI and more prevalence of DM, CVD, and chronic lung disease. Regarding medication use, the high AGR group had the lower proportion of prescriptions of ESA and CCB, and a higher proportion of prescriptions of pentoxifylline compared with the other groups. There were significant differences in most of the laboratory measurements among the three groups, except for cholesterol level.

### 3.2. Clinical Outcomes among the Study Patients

**Figure 1:**
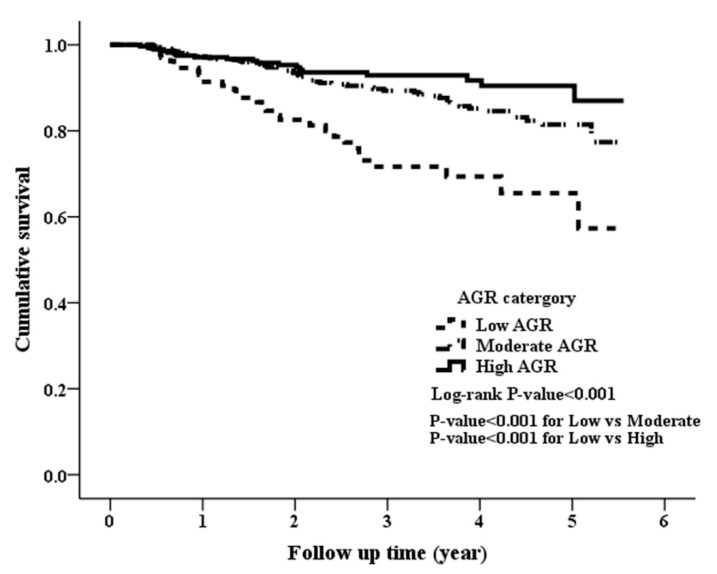
Kaplan-Meier curve of overall patient survival according to the AGR groups (log-rank test, *p* < 0.001).

During a median follow-up duration of 2.44 years, 108 (11.3%) deaths were recorded among the 956 patients. A total of 31 (22.46%), 58 (10.84%), and 19 (6.71%) patients died in the low, moderate, and high AGR groups, respectively (*p* < 0.001). The Kaplan-Meier estimate of survival was shown in Figure 1, illustrating there was a significant difference in overall survival among the three groups (log-rank *p* < 0.001). The low AGR group had the worst overall survival while the high AGR group had the best overall survival.

**Figure 2:**
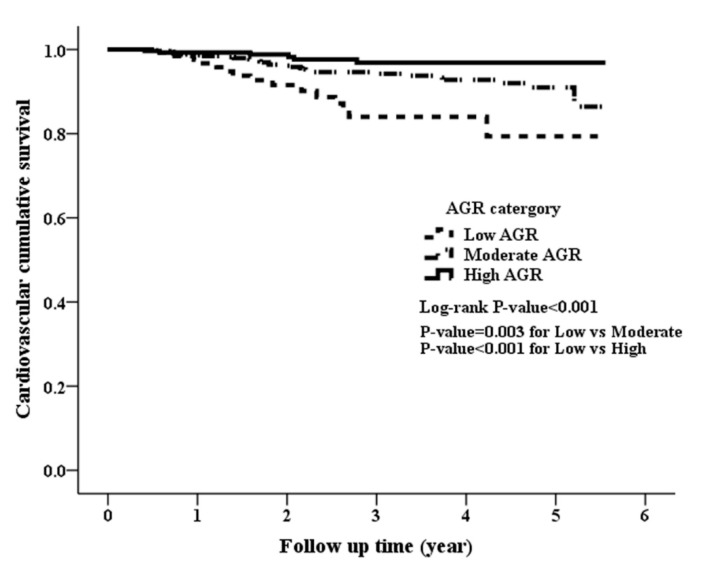
Kaplan-Meier curve of cumulative survival free of cardiovascular disease-related mortality according to the AGR groups (log-rank test, *p* < 0.001).

Of the 108 deaths, 50 (46.3%) patients died from CVD. There was also a significant difference in CVD mortality rate among the three groups, with 15 (10.87%), 29 (5.42%), and 6 (2.12%) in the low, moderate, and high AGR groups, respectively (*p* = 0.001). Figure 2 illustrated the Kaplan-Meier analysis of CVD survival with a log-rank *p* < 0.001, indicating that the low AGR group had the worst cardiovascular survival and the high AGR group had the best cardiovascular survival.

### 3.3. Adjusted Associations of AGR Groups with Clinical Outcomes

In the crude Cox models, the moderate and high AGR groups were associated with a reduced risk of all-cause and CVD mortality compared with the low AGR group (Table 2). In adjusted model 1, the moderate AGR group was associated with HRs of 0.57 (95% CI = 0.36–0.90, *p* = 0.016) and 0.52 (95% CI = 0.28–0.98, *p* = 0.043) for all-cause and CVD mortality compared with the low AGR group, respectively. The high AGR group was associated with HRs of 0.49 (95% CI = 0.27–0.90, *p* = 0.021) and 0.27 (95% CI = 0.1–0.74, *p* = 0.01) for all-cause and CVD mortality compared with the low AGR group, respectively.

The maximum standardization difference between the AGR groups before and after IPW was shown in Table 3. After IPW, the covariates with a maximum standardization difference >0.1 included sex, CVD, and the use of pentoxifylline, which were all incorporated as adjustments in Model 2. In adjusted Model 2 (IPW Cox model), the adjusted HRs for the moderate AGR group versus the low AGR group were 0.72 (95% CI = 0.54–0.97, *p* = 0.028) and 0.78 (95% CI = 0.52–1.18, *p* = 0.237) for all-cause and CVD mortality, respectively. The adjusted HRs for the high AGR group versus the low AGR group were 0.72 (95% CI = 0.52–0.99, *p* = 0.046) and 0.46 (95% CI = 0.27–0.8, *p* = 0.006) for all-cause and CVD mortality, respectively.

### 3.4. Sensitivity Analysis

Two levels of sensitivity analyses were constructed as shown in Table 2. We repeated the adjusted model 1 and 2 to maximize the predictive value of the AGR for mortality risk by treating the AGR as a continuous variable. In the model 1 adjustment, the HRs for every 1-unit increase in AGR were 0.27 (95% CI = 0.13–0.61, *p* = 0.001) and 0.21 (95% CI = 0.07–0.67, *p* = 0.009) for all-cause and CVD mortality, respectively. In the model 2 adjustment, the HRs for every 1-unit increase in AGR were 0.49 (95% CI = 0.32–0.78, *p* = 0.002) and 0.37 (95% CI = 0.19–0.72, *p* = 0.003) for all-cause and CVD mortality, respectively.

### 3.5. Stratified Analyses

Table 4 showed the adjusted associations of AGR with risk of all-cause and CVD mortality in stratified subgroups. All the *p* values for the interaction were >0.05, indicating no significant interaction effects between the AGR and those pre-specified subgroups. Thus, the association of the AGR with all-cause and CVD mortality did not differ by sex, age, and the presence or absence of DM, hypertension, gout, chronic lung disease, and CVD. The results of the stratified analysis were consistent with the preliminary results.

## 4. Discussion

Low serum albumin was reported to be predictive of poor survival in patients with CKD [7]. To the best of our knowledge, this is the first work showing the prognostic value of the AGR in terms of overall and cardiovascular mortality in patients with pre-dialysis CKD. In the present study, we showed that the AGR was associated with patient mortality of all causes as well as CVD, independent of a variety of clinically confounding factors. Patients with a higher AGR level were associated with a reduced risk of overall and cardiovascular mortality in comparison to patients with a lower AGR level. The mortality risk was further attenuated with the increasing AGR as demonstrated by the adjusted risk of death which showed a gradual decrease from the moderate AGR group to the high AGR group compared with the low AGR group. In addition, the AGR as a continuous variable remained a significant predictor of patient survival, further strengthening our findings.

Albumin is commonly regarded as a biological marker for assessing nutritional status. The etiologies of hypoalbuminemia include malnourishment, hepatic impairment, decreased hepatic synthesis of albumin due to interleukin (IL)-1, IL-6, and tumor necrosis factor (TNF)-α, and protein loss via the kidney or gastrointestinal tract [13]. Irrespective of its causes, hypoalbuminemia powerfully predicts morbidity and mortality [14,15]. Low serum albumin negatively affects patient prognosis through several mechanisms. Albumin has an antioxidant effect against carcinogens, and stabilizes cell growth and DNA replication [16]. On the other hand, hypoalbuminemia may indicate malnutrition, which weakens several human immune defense barriers and increases the susceptibility to sepsis. Low serum albumin was more greatly attributed to systemic inflammation than malnutrition in dialysis patients [17]. Interestingly, the beneficial effects of albumin are further explained as they are associated with the mobilization of polyunsaturated fatty acids (PUFA). A sufficient amount of albumin is likely to mobilize PUFA from the liver and aid in the formation of lipoxins, resolvins, and protectins, which are anti-inflammatory lipids [18]. Therefore, hypoalbuminemia may tend to proinflammatory status, leading to increased morbidity and mortality.

Kidney function has been shown to be inversely correlated with inflammation. Inflammation is known to be an important constituent of CKD, and it is also closely implicated with the development of cardiovascular events in CKD patients [19]. The causes of inflammatory activation in CKD include oxidative stress, periodontal disease, decreased cytokines elimination, vitamin D deficiency, and so on [20]. In addition, multiple factors can contribute to inflammatory activation in dialysis patients, including impurities and microbiological quality in dialysis solution for hemodialysis modality, as well high-glucose solution and peritonitis for peritoneal dialysis modality [21,22,23]. The association of C-reactive protein (CRP) with mortality was first reported in hemodialysis patients by Bergstrom et al. in 1995 [24]. The constant conclusions were also drawn by subsequent investigations showing CRP as a significant predictor in patients with CKD/ end-stage renal disease [25,26]. Growing amounts of evidence supporting the role of inflammation in CKD leads to our perception of inflammation as an established risk factor for morbidity and mortality. Interestingly, serum amyloid-A was recently reported as a superior predictor of mortality compared to CRP, leukocytes, fibrinogen, and erythrocyte sediment rate in patients on peritoneal dialysis [27]. Globulin is the major part of non-albumin protein in the serum and consists of many pro-inflammatory proteins, such as immunoglobulins, complements, and C-reactive protein. High globulin levels are thought to reflect an inflammation and/or host immune response [28]. Subsequently, all of these results related to nutritional and inflammatory status led us to conclude that the predictive value of the AGR is enhanced in the CKD population.

Albumin, a nutritional parameter, is affected by the inflammation, so albumin is expected to be negatively interrelated with inflammation. As with other studies, the AGR in the present study is derived from the ratio of albumin to non-albumin total protein, so it is reflective of both nutrition and inflammation in CKD patients. Evidence supporting the prognostic value of the AGR recently emerged, with the majority of the investigations in cancer patients. Preoperative AGR can act as a valuable predictor of prognosis in patients with colorectal cancer, nasopharyngeal cancer, lung cancer, and breast cancer [8,9,10,11]. Furthermore, low AGR was associated with cancer incidence and mortality in generally healthy adults [29]. The result of our work in finding that the AGR was predictive of patient survival reminds us of protein-energy wasting (PEW), which describes the decreased body stores of protein and energy resulting from under-nutrition and inflammation, and is associated with poor outcomes in CKD patients. The prevalence of PEW in non-dialyzed stage 3–5 CKD patients was estimated to range between 9% and 18% [30,31,32]. The pathophysiological mechanisms linking PEW to adverse outcomes are not fully elucidated and a variety of metabolic, hormonal, nutritional, and immunological derangements are suggested to play a vital role for the higher infection rates and mortality risk [33]. Treatments of PEW in CKD include anabolic steroids, growth hormone, physical exercise, and nutritional supplements, administered enterally, as well as intradialytic parenteral nutrition [34]. Appetite stimulants (e.g., megestrol acetate and ghrelin) and anti-inflammatory agents (e.g., pentoxyfylline, etanercept, and IL-1 receptor antagonist) are emerging as novel therapies [34]. However, large randomized clinical trials are warranted to test the impact of the above mentioned interventions not only in terms of improvements in nutritional markers, but also on mortality and morbidity. Nonetheless, we believe that AGR may serve as a promising surrogate biomarker for the detection and identification of PEW in CKD patients, and this issue can be an interesting topic for further research.

The results of our work suggested that the AGR is predictive of mortality risk in stage 3–5 CKD patients. Nutritional status and chronic inflammation play a major role in predicting mortality risk in CKD patients. Based on our work, the AGR should be assessed in patients with stage 3–5 CKD. Patients with a low AGR should be closely monitored to clarify whether there is malnourishment, high inflammatory reaction, or both. Proper nutritional counseling with support and potential anti-inflammatory therapies for those candidate patients might hopefully improve their outcomes. However, a different AGR cut-off value may be determined in a particular cohort of CKD patients and distinct results may be concluded with respect to survival comparisons; therefore, an optimal AGR value for CKD patients is required for generalization to the whole CKD population.

There are several limitations for a retrospective study such as ours. First, our research lacked the measurement of CRP, tumor necrosis factor, and cytokine levels since they weren’t routinely checked for our patients. Therefore, their associations with the AGR and survival status could not be examined. Instead, white blood cell (WBC) count was adjusted as an inflammatory index in the analysis. However, it needs to be determined whether the significant association between the AGR and clinical outcomes would remain if the Cox regression models incorporated other known inflammatory biomarkers. We also cannot compare the predictive value of the AGR with other unmeasured inflammatory parameters, such as IL-1, IL-6, and TNF-α. Second, we only took a single determination of the AGR into analyses. The variations in the AGR over time may be reflective of the changes in nutritional and inflammatory status in response to treatments, and may influence the clinical outcomes in a positive or negative way. Third, albumin is exclusively synthesized in the liver, so the level of liver reserve could influence the patients’ clinical outcomes significantly. We cannot mitigate the impact of the liver functional reserve because the serum levels of total bilirubin, glutamic-pyruvic transaminase, glutamic-oxalocetic transaminase, and international normalized ratio were not available in our retrospective data.

In conclusion, the AGR is a significant biomarker predicting overall and cardiovascular mortality risk independent of various important factors amongst stage 3–5 CKD patients. Moreover, this relationship between AGR and mortality was further enhanced by demonstrating a consistent result with the AGR as a continuous variable. Since the assessment of the AGR is inexpensive, accessible, easy to test, and has standardized measurement criteria worldwide, the AGR is considered as a novel index to better stratify those patients who may benefit more from the limited medical resources. Our findings need to be confirmed by a multi-institutional study with a prospective design. Whether therapeutic strategies to those high-risk patients recognized by the AGR can improve their long-term clinical outcomes is of substantial interest and will be the subject of research in the future.

## Figures and Tables

**Table 1:** Baseline characteristics of the study population by the AGR groups.

	Low AGR	Moderate AGR	High AGR	*p*-Value
	(AGR < 1.0)	(1.0 ≤ AGR < 1.3)	(AGR ≥ 1.3)	
**Number of Patients**	138	535	283	--
Sex, men	65 (47.1%)	276 (51.59%)	188 (66.43%)	<0.004
Age (years)	69.7 ± 12.8	68.6 ± 12.3	65.4 ± 13.6	<0.001
Body mass index (kg/m^2^)	26.34 ± 5.23	25.34 ± 4.24	25.00 ± 4.1	0.011
Smoking				
Never	89 (64.49%)	371 (69.35%)	176 (62.19%)	0.103
Current	15 (10.87%)	60 (11.21%)	37 (13.07%)	0.694
Ever	34 (24.64%)	104 (19.44%)	70 (24.73%)	0.147
Alcohol				
Never	108 (78.26%)	419 (78.32%)	194 (68.55%)	0.006
Current	6 (4.35%)	39 (7.29%)	40 (14.13%)	0.001
Ever	24 (17.39%)	77 (14.39%)	49 (17.31%)	0.461
CKD stages				
Stage 3	32 (23.19%)	238 (44.49%)	164 (57.95%)	<0.001
Stage 4	62 (44.93%)	183 (34.21%)	79 (27.92%)	<0.001
Stage 5	44 (31.88%)	114 (21.31%)	40 (14.13%)	<0.001
**Comorbidity Disease**				
Gout	32 (23.19%)	141 (26.36%)	81 (28.62%)	0.489
Hypertension	107 (77.54%)	395 (73.83%)	201 (71.02%)	0.354
Diabetes mellitus	86 (62.32%)	270 (50.47%)	105 (37.1%)	<0.001
Cardiovascular disease	70 (50.72%)	208 (38.88%)	82 (28.98%)	<0.001
Chronic Lung disease	27 (19.57%)	72 (13.46%)	31 (10.95%)	0.053
**Medication use**				
NSAID	4 (2.9%)	17 (3.18%)	13 (4.59%)	0.526
ACEI/ARB	83 (60.14%)	310 (57.94%)	152 (53.71%)	0.368
Statin	66 (47.83%)	244 (45.61%)	120 (42.4%)	0.523
Erythropoiesis-stimulating agents	23 (16.67%)	68 (12.71%)	20 (7.07%)	0.008
Vitamin D	6 (4.35%)	17 (3.18%)	11 (3.89%)	0.754
Calcium supplement	17 (12.32%)	40 (7.48%)	17 (6.01%)	0.071
Calcium channel blockers	79 (57.25%)	308 (57.57%)	134 (47.35%)	0.016
Pentoxifylline	72 (52.17%)	308 (57.57%)	183 (64.66%)	0.032
**Laboratory data**				
AGR	0.8 (0.7–0.9)	1.2 (1.1–1.2)	1.5 (1.4–1.6)	<0.001
Albumin (g/dL)	3.2 (2.8–3.4)	3.7 (3.5–3.9)	4 (3.8–4.2)	<0.001
Blood urea nitrogen (mg/dL)	40 (29–53)	32 (25–50)	28 (23–39)	<0.001
Calcium (mg/dL)	8.9 (8.5–9.2)	9.2 (8.9–9.4)	9.3 (9–9.5)	<0.001
Cholesterol (mg/dL)	170.5 (142–207)	174 (148–200)	177 (149–204)	0.625
Creatinine (mg/dL)	2.76 (2.01–4.38)	2.18 (1.66–3.34)	1.95 (1.67–2.75)	<0.001
eGFR (mL/min/1.73 m^2^)	20.82 (11.67–28.44)	27.75 (16.38–36.13)	33.39 (21.29–39.39)	<0.001
Hemoglobin (g/dL)	9.95 (9.1–11.2)	11.1 (9.7–12.5)	12.1 (10.5–13.5)	<0.001
HbA1c (%)	6.2 (5.7–7.3)	6 (5.6–6.9)	5.8 (5.5–6.3)	<0.001
Potassium (mEq/L)	4.3 (3.8–4.7)	4.4 (4.1–4.8)	4.4 (4.1–4.7)	0.024
Sodium (mEq/L)	138 (136–139)	139 (137–140)	139 (138–140)	<0.001
Phosphate (mg/dL)	4.3 (3.7–4.7)	4 (3.6–4.6)	3.8 (3.5–4.3)	<0.001
Triglyceride (mg/dL)	112.5 (81–174)	119 (87–171)	131 (95–188)	0.042
UPCR (mg/g)	1822.05 (584.5–5290.9)	943.3 (269.4–2215.5)	430 (137.2–1403.5)	<0.001
Uric Acid (mg/dL)	7.8 (6.4–8.7)	7.5 (6.5–8.5)	7.2 (6.4–8.1)	0.014
WBC (× 10^3^/μL)	7.3 (5.9–9)	6.7 (5.6–8)	6.4 (5.4–7.8)	0.002

Values are expressed as mean ± SD, median and interquartile range, or number (percentage).Abbreviations: AGR, albumin-globulin ratio; ACE inhibitor, angiotensin-converting enzyme inhibitor; ARB, angiotensin II receptor blocker; eGFR, estimated glomerular filtration rate; HbA1c, hemoglobin A1c; UPCR, urine protein-to-creatinine Ratio; WBC, white blood cell; NSAID, non-steroidal anti-inflammatory drug.

**Table 2:** Univariate and multivariate Cox regression models of all-cause and CVD-related mortality for AGR groups.

AGR Category †: Low AGR Group: AGR ≤ 1.0 (as Reference Group); Moderate AGR Group: 1.1 ≤ AGR < 1.3; High AGR Group: AGR ≥ 1.3		All-Cause Mortality		CVD Mortality	
		Hazard Ratio (95% CI)	*p*-Value	Hazard Ratio (95% CI)	*p*-Value
(A)Crude model	Low	1		1	
	Moderate	0.56 (0.43,0.74)	<0.001	0.61 (0.41,0.91)	0.014
	High	0.46 (0.34,0.63)	<0.001	0.27 (0.16,0.46)	<0.001
Model 1	Low	1		1	
	Moderate	0.57 (0.36,0.9)	0.016	0.52 (0.28,0.98)	0.043
	High	0.49 (0.27,0.9)	0.021	0.27 (0.1,0.74)	0.010
Model 2	Low	1			
	Moderate	0.72 (0.54,0.97)	0.028	0.78 (0.52,1.18)	0.237
	High	0.72 (0.52,0.99)	0.046	0.46 (0.27,0.8)	0.006
**(B) sensitivity tests**					
(i) AGR as a continuous variable in model 1	--	0.27 (0.13,0.61)	0.001	0.21 (0.07,0.67)	0.009
(ii) AGR as a continuous variable in model 2	--	0.49 (0.32,0.78)	0.002	0.37 (0.19,0.72)	0.003

Crude model: crude hazard ratio (HR) of AGR category. Estimated hazard ratios were derived from Cox’s proportional hazard models. Model 1 adjusted for AGR categories, laboratory data, and propensity score. Model 2 was performed using inverse probability of group-weighted (IPW) Cox model, in which the covariates included AGR categories, laboratory data and the variables in Table 3 with maximum standardization difference >0.10 were selected for adjustment model. † Study patients were classified into low, moderate, and high AGR categories based on similar magnitudes of hazard. Abbreviations: AGR, albumin-globulin ratio; CVD, cardiovascular disease.

**Table 3:** Covariate balance between AGR categories before and after inverse probability of treatment weighted standardization.

	Maximum Standardization Difference Between Groups	
	Before IPW ^a^ (%)	After IPW ^a^ (%)
Gender, male	0.259	0.180 *
Age (years)	0.224	0.035
Body mass index	0.205	0.080
Smoking	0.101	0.016
Alcohol	0.113	0.071
**Comorbidity disease**		
Gout	0.082	0.032
Hypertension	0.099	0.036
Diabetes mellitus	0.336	0.076
Cardiovascular disease	0.299	0.109 *
Chronic Lung disease	0.167	0.094
**Medication use**		
NSAID	0.061	0.012
ACEI/ARB	0.086	0.053
Statin	0.073	0.059
Erythropoiesis-stimulating agents	0.199	0.047
Vitamin D	0.042	0.040
Calcium supplement	0.157	0.021
Calcium channel blockers	0.137	0.084
Pentoxifylline	0.169	0.119*

^a^ Inverse probability of group-weighting (IPW) was estimated by the propensity of group from the generalized boosted regression. Abbreviations: NSAID, non-steroidal anti-inflammatory drug; AGR, albumin-globulin ratio; ACE inhibitor, angiotensin-converting enzyme inhibitor; ARB, angiotensin II receptor blocker. * The maximum standardization differences after IPW were more than 0.1.

**Table 4:** Adjusted associations of AGR with risk of all-cause and CVD mortality in pre-specified subgroups.

Subgroup	All-Cause Mortality		CVD Mortality	
	Hazard Ratio (95% CI)	P Interaction	Hazard Ratio (95% CI)	P Interaction
Male	0.24(0.13,0.44)	0.918	0.13(0.03,0.64)	0.929
Female	0.33(0.09,1.15)		0.17(0.03,1.01)	
Age < 65 (years)	0.08(0.01,0.55)	0.793	0.35(0.01,10.78)	0.834
Age ≧ 65 (years)	0.50(0.20,1.22)		0.25(0.07,0.93)	
DM	0.36(0.12,1.09)	0.157	0.314(0.06,1.61)	0.742
No DM	0.21(0.07,0.66)		0.112(0.02,0.62)	
Hypertension	0.23(0.08,0.64)	0.560	0.113(0.03,0.51)	0.252
No hypertension	0.30(0.08,1.15)		0.339(0.06,1.84)	
Gout	0.56(0.11,2.97)	0.459	0.363(0.03,4.39)	0.792
No gout	0.16(0.07,0.39)		0.153(0.04,0.56)	
Chronic lung disease	0.35(0.09,1.42)	0.405	1.448(0.14,15.26)	0.130
No chronic lung disease	0.24(0.10,0.62)		0.116(0.03,0.42)	
CVD	0.47(0.17,1.30)	0.108	0.37(0.08,1.7)	0.895
No CVD	0.17(0.05,0.58)		0.125(0.02,0.87)	

Hazard ratio was calculated using Cox proportional regression analyses adjusted for laboratory data and propensity score.

## Supplementary Materials

The following are available online at https://www.mdpi.com/2077-0383/8/11/1991/s1, Figure S1: The study population was classified as low, moderate, or high AGR groups based on similar magnitudes of mortality risk.
