# Supplementary material for: Association between Albumin–Globulin Ratio and Mortality in Patients with Chronic Kidney Disease
Source: J Clin Med. 2019 Nov 15;8(11):1991. doi: 10.3390/jcm8111991 (PMC6912628; doi:10.3390/jcm8111991)
Supplement: Supplementary file 1 [file jcm-08-01991-s001.pdf]

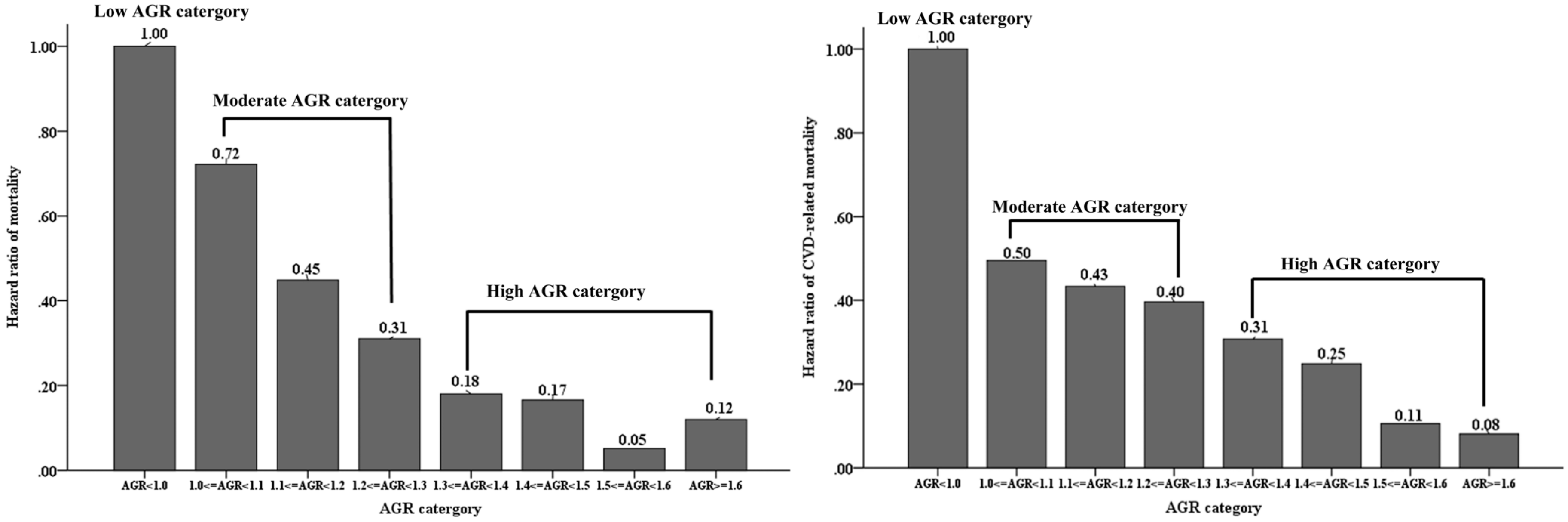

Figure S1. The study population was classified as low, moderate, or high AGR groups based on similar magnitudes of mortality risk.
